# Fetal mortality and the challenges for women's health care in Brazil

**DOI:** 10.11606/S1518-8787.2019053000714

**Published:** 2019-01-18

**Authors:** Patrícia de Sá Barros, Érika Carvalho de Aquino, Marta Rovery de Souza

**Affiliations:** I Universidade Federal de Goiás, Regional Jataí. Unidade Acadêmica Especial Ciências da Saúde. Curso de Fisioterapia. Jataí, GO, Brasil; II Universidade Federal de Goiás. Instituto de Patologia Tropical e Saúde Pública. Programa de Pós-Graduação em Medicina Tropical e Saúde Pública. Goiânia, GO, Brasil; III Universidade Federal de Goiás. Instituto de Patologia Tropical e Saúde Pública. Departamento de Saúde Coletiva. Goiânia, GO, Brasil

**Keywords:** Fetal Mortality, trends, Perinatal Mortality, trends, Fetal Death, Mortality Registries, Time Series Studies, Mortalidade Fetal, tendências, Mortalidade Perinatal, tendências, Morte Fetal, Registros de Mortalidade, Estudos de Séries Temporais

## Abstract

**OBJECTIVE:**

To establish a historical series of fetal mortality in Brazil and regions between 1996 and 2015, identifying its behavior and trend.

**METHODS:**

A descriptive study on cases of fetal deaths in Brazil and in each region reported from 1996 to 2015, registered in DATASUS and classified by ICD-10. Maternal age and schooling, duration of gestation and type of delivery were considered. We calculated the fetal mortality rate between 1996 and 2015 to build historical series.

**RESULTS:**

The time series shows a steady chart of the fetal mortality rate (FMR) from 2000 in Brazil and in all regions. The country's fetal mortality rate rose from 8.19 in 1996 to 9.50 per 1,000 births in 2015. There was an increasing trend in fetal deaths whose root cause appears in chapter XVII of ICD-10 in Brazil and in all regions. Deaths from Chapter XVI causes showed a trend of increase only in the Northeast region, while other basic causes showed a trend of increase in the Southeast and South regions. In the Brazilian scope, there was an increasing trend of fetal deaths in mothers in the 10-14 and 25-44 years age groups. In Brazil and in all regions, there was an increase of the FMR in women with more than 8 years of schooling. Fetal deaths predominated between 28 and 36 weeks of gestation, with a growing trend in Brazil and all regions, except in the South (steady). Vaginal delivery prevailed, with a steady trend, while cesarean sections showed an increasing trend in Brazil and in all regions.

**CONCLUSIONS:**

The quality of information about fetal deaths, investments in research committees, and improvement in the quality of prenatal care should be prioritized to enable more effective coping and to reduce the fetal mortality rate in Brazil.

## RESUMO

**OBJETIVO:**

Traçar uma série histórica da mortalidade fetal no Brasil e regiões entre 1996 e 2015, identificando seu comportamento e tendência.

**MÉTODOS:**

Estudo descritivo sobre casos de óbitos fetais no Brasil e em cada região notificados de 1996 a 2015, registrados no Datasus e classificados pela CID-10. A idade e escolaridade da mãe, duração da gestação e tipo de parto foram considerados. Foi realizado o cálculo da taxa de mortalidade fetal entre 1996 e 2015 para construção das séries históricas.

**RESULTADOS:**

A série temporal apresenta um quadro estacionário na taxa de mortalidade fetal a partir de 2000 no Brasil e em todas as regiões. A taxa de mortalidade fetal do país passou de 8,19 em 1996 para 9,50 por 1.000 nascimentos em 2015. Houve tendência crescente dos óbitos fetais cuja causa básica consta no capítulo XVII da CID-10 no Brasil e em todas as regiões. Óbitos por causas do capítulo XVI apresentaram tendência de aumento somente na região Nordeste, enquanto outras causas básicas mostraram tendência de aumento nas regiões Sudeste e Sul. No âmbito brasileiro, houve tendência crescente de óbitos fetais em mães nas faixas etárias de 10–14 anos e 25–44 anos. No Brasil e em todas as regiões, houve aumento nas mulheres com mais de oito anos de escolaridade. Os óbitos fetais predominaram entre a 28ª e a 36ª semana de gestação, com tendência crescente no Brasil e todas as regiões, exceto no Sul (estacionário). O tipo de parto predominante foi vaginal, com tendência estacionária, enquanto as cesarianas apresentaram tendência crescente no Brasil e em todas as regiões.

**CONCLUSÕES:**

A qualidade da informação sobre os óbitos fetais, investimentos nos comitês de investigação e melhora na qualidade do pré-natal devem ser priorizados para possibilitar um enfrentamento mais efetivo e diminuir a taxa de mortalidade fetal no Brasil.

## INTRODUCTION

The World Health Organization (WHO) defines fetal death, according to the International Statistical Classification of Diseases and Related Health Problems – 10th Revision (ICD-10), as death prior to the complete expulsion from its mother of a product of conception, irrespective of the duration of pregnancy; indicated by the fact that the fetus does not breathe or show any other evidence of life^1^ ^,^ ^2^.

One of the indicators of quality of care provided to pregnant women and childbirth is the fetal mortality rate (FMR), expressed as the number of fetal deaths occurring from the 22^nd^ complete week of gestation or 154 days, with fetuses weighing at least 500 g or height from 25 cm, per thousand total births in the resident population in a given geographical area and year^3^.

In the period from 1995 to 2009, the worldwide FMR fell by 14.0%, from 22.1 to 18.9 per 1,000 total births^4^. In 2011, it was estimated that approximately 2.6 million fetal deaths occurred globally in 2009^4^ ^-^ ^6^. In 2014, the Action Plan for Newborns was launched, a movement to prevent fetal and maternal mortality, predicting by 2030 a FMR of 12 or fewer fetal deaths per 1,000 births in all countries and actions to address the disparities found^6^. A systematic analysis indicated that the annual rate of reduction of global fetal deaths in the 2000–2015 period was 2.0 fetal deaths per 1,000 births^6^.

Despite its relevance, the indicator was not included by the United Nations as one of the Millennium Development Goals, thus being ignored and invisible in global policy agendas^6^ ^-^ ^9^.

In 2016, 1.7 million fetal deaths were recorded worldwide, a decrease of 65.3% since 1970 due to the increase in the number of live births, which increased from 114.1 million in 1970 to 128.8 million in 2016. Fetal death rates decreased by 68.4% from 41.5 in 1970 to 13.1 deaths per 1,000 live births in 2016. The lowest FMR in 2016 was in Finland (1.11 per 1,000 births) and the highest in Sudan (43.4 per 1,000 live births). Regionally, stillbirth rates were higher in countries in central sub-Saharan Africa, which exceeded 23 deaths per 1,000 births in 2016. The rates were highly variable across South and Southeast Asia, from 3.5 deaths per 1,000 births in Malaysia to 25.9 deaths per 1,000 births in Pakistan. Only six Western European countries had a fetal death rate below 1.5 per 1,000 in 2016, a feat that was not achieved by any country in the Americas. In 114 of 195 countries, fetal death rates declined in the last decades, more rapidly in the years after 2000 than in the 1990-2000 decade. In Brazil, the rate was 5.3 (4.9 to 5.8) fetal deaths per 1,000 births from 2000 to 2016^10^.

In Brazil, government public policies related to women's health care were limited to the concern with the maternal-child group. Only in 1983 did the Ministry of Health implement the *Programa de Assistência Integral à Saúde da Mulher* (PAISM – Integral Care Program to Women's Health), which considers women as an active subject in a social context, encompassing women's care in clinical and gynecological changes; in the control of prenatal care, delivery and puerperium; in sexually transmitted diseases; in cervical uterine and breast cancer; in conception and contraception, from adolescence to old age^11^. In 2004, under the gender focus, the Brazilian Ministry of Health progressed with the *Política Nacional de Atenção Integral à Saúde da Mulher* (PNAISM – National Policy of Integral Attention to Women's Health), which aimed at improving the integrality and promotion of women's health, sexual and reproductive rights, obstetric care, family planning, attention to abortion, and the fight against domestic and sexual violence^12^.

After almost thirty years, an innovative Ministry of Health strategy through the Health Surveillance Secretariat, under the Unified Health System (SUS), aims to promote the implementation of a new model of health care for women and children. Instituted by Administrative Rule 1459, dated June 24, 2011, the *Rede Cegonha* includes a package of actions to structure and organize maternal and child health care, ensuring the right to reproductive planning, humanized care in pregnancy, childbirth and puerperium and the safe birth of children, as well as their healthy growth and development^13^ ^,^ ^14^.

However, the low visibility of the subject, despite its great importance, reinforces the need for studies to identify the occurrence of fetal deaths and contribute to the planning of specific actions that reduce the FMR in Brazil. This study was designed to establish a historical series of fetal mortality in Brazil and in its five regions between 1996 and 2015, identifying its behavior and trend and seeking to support actions for women's health care that reduce fetal mortality.

## METHODS

This is a descriptive, retrospective study based on secondary data on fetal deaths recorded in the Informatics Department of the Brazilian National Health System (DATASUS) of the Ministry of Health. Fetal deaths reported from 1996 to 2015 in Brazil and in each region, present in the health information system (TABNET) and classified by ICD-10, were included. Data unrelated to the proposed period of study were excluded.

This period was selected for the availability of data on mortality and population in DATASUS, and it begins in 1996 because it was the year of publication of ICD-10.

Information on live births and fetal deaths are public domain and were collected from the Live Birth Information System (SINASC) and the Mortality Information System (SIM) during August 2017.

In 2016 Brazil had an estimated population of 204,450,380 people in five regions: North (17,472,646 people), Northeast (56,560,034 people), Southeast (85,745,427 people), South (29,230,070 people) and Midwest (15,442,203 people)^15^.

Data on fetal deaths were obtained at http://datasus.saude.gov.br/ ^16^, following the path: Access to Information > Health Information (TABNET) > Vital Statistics > Mortality – 1996 to 2015, by CID-10 > Fetal deaths > Geographic coverage > Brazil by Region and Federation Unit. In the line the year of death was selected, in the column the region of Brazil and in the content the deaths by place of residence.

The following variables were considered:

- Region: all categories (North, Northeast, Southeast, South, Midwest);
- ICD-10 chapter (cause of death): 21 Chapters^a^;
- Maternal age: less than 10 years, 10–14 years, 15–19 years, 20–24 years, 25–29 years, 30–34 years, 35–39 years, 40–44 years, 45–49 years, 50–54 years, 55–59 years, 60–64 years, unknown;
- Maternal schooling: none, 1–3 years, 4–7 years, 8–11 years, 12 or more, elementary school, high school, higher education, n.a.;
- Length of gestation (number of weeks): less than 22 weeks, 22–27 weeks, 28–31 weeks, 32–36 weeks, 37–41 weeks, 42 weeks or more, n.a.;
- Type of delivery: vaginal, cesarean, forceps or other (until 1995), n.a.;
- Birth weight: 500–999 g, 1,000–1,499 g, 1,500–2,499 g, 2,500–2,999 g, 3,000–3,999 g, 4,000 g or more.

The existence of overlapping or different categories is due to changes in forms over time.

### Estimation Method

For estimation of the number of deaths between 1996 and 2015, historical series were developed for the period.

The fetal mortality rate (FMR) was used, which includes: number of fetal deaths (22 weeks of gestation or more)^b^ from mothers residing in Brazil or regions studied × 1,000 / total number of births of resident mothers (live births plus fetal deaths at 22 weeks of gestation or more).

The study was not submitted to the Ethics Committee in Research on Human Beings because it was about public information.

To analyze the trend of death rates, we used the Prais-Winsten method for generalized linear regression, allowing comparison of the different time series under study. This method was preferred to simple linear regression because it is especially designed for data that can be influenced by serial autocorrelation, which often occurs in population data measures. By means of the Prais-Winsten regression we found the value of b, referring to the slope of the line. The statistical significance was p ≤ 0.05.

Data were tabulated with TabNet software. Microsoft^®^ Excel was used to organize the database. Statistical analyses were performed with Stata 13.0.

## RESULTS

In the 1996-2015 period, 553,718 thousand fetal deaths occurred in Brazil, 59,205 (11.0%) in the North, 175,591 (32.0%) in the Northeast, 218,858 in the Southeast (40.0%), and 63,176 in the South (11.0%) and 36,888 (7.0%) in the Midwest.

The time series depicted in the Figure shows an unchanging FMR from 2000 in Brazil (b = 0.602, p = 0.849) and in all regions: North (b = 2.870, p = 0.630), Southeast (b = 0.717, p = 0.839), South (b = 0.520, p = 0.849), Midwest (b = 2.750, p = 0.434) and Northeast (b = 9.770, p = 0.076). The FMR in Brazil increased from 8.19 in 1996 to 9.50/1,000 births in 2015, considered a steady trend.

**Figure:**
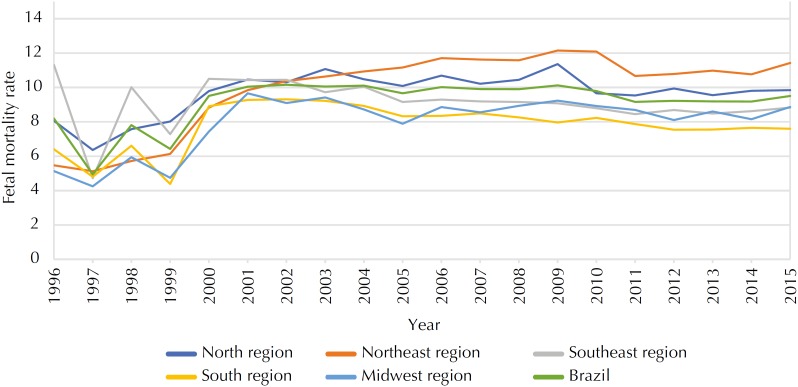
Trend of fetal mortality in Brazil and regions, 1996–2015.

Table 1 shows the basic causes of deaths according to ICD-10 chapters. There was an increasing trend of fetal deaths whose basic cause pertained to chapter XVI (certain conditions originating in the perinatal period) in the Northeast region (b = 0.017, p = 0.019). In Brazil and other regions, this trend was steady. Concerning Chapter XVII (Congenital malformations, deformations and chromosomal abnormalities), the data showed a trend of increase in Brazil and in all regions. The other basic causes, included in chapters I, II, III, IV, V, VI, VII, VIII, IX, X, XI, XII, XIII, XIV, XIX, XX, XXI and XXII showed a trend of fetal death increase in the Southeast (b = 0.034, p = 0.006) and South (b = 0.032, p = 0.020). These chapters were analyzed together because they represent a small proportion of the total number of deaths.

**Table 1:** Trend of fetal death numbers according to ICD-10 chapters. Brazil and regions, 1996–2015.

Regions	Chapter XVI			Chapter XVII			Other causes			Total		
	b	p	Trend	b	p	Trend	b	p	Trend	b	p	Trend
North	0.005	0.220	Steady	0.018	0.001	Increasing	-0.003	0.924	Steady	0.005	0.177	Steady
Northeast	0.017	0.019	Increasing	0.027	0.001	Increasing	-0.007	0.773	Steady	0.017	0.016	Increasing
Southeast	-0.001	0.805	Steady	0.011	0.004	Increasing	0.034	0.006	Increasing	0.000	0.874	Steady
South	-0.005	0.468	Steady	0.015	0.029	Increasing	0.032	0.020	Increasing	0.005	0.283	Steady
Midwest	-0.008	0.373	Steady	0.022	0.006	Increasing	0.018	0.239	Steady	0.011	0.039	Increasing
Brazil	0.004	0.371	Steady	0.022	0.000	Increasing	0.042	0.129	Steady	0.005	0.156	Steady

Table 2 shows the trends in the number of deaths in relation to maternal age groups. In Brazil, there was an increasing trend in the number of fetal deaths in mothers aged 10–14 (b = 0.012, p = 0.001), 25–29 (b = 0.011, p = 0.014), 30–34 (b = 0.015, p ≤ 0.001), 35–39 (b = 0.014, p = 0.004) and 40–44 (b = 0.013, p = 0.016) years, as well as a steady curve in the number of mothers in the age groups 15–19 (b = 0.004, p = 0.363) 20–24 (b = 0.005, p = 0.361) and 45–49 years (b = 0.007, p = 0.284). In particular, the 15–19 and 20–24 years age groups showed steady charts in Brazil and in all regions. The 30–34 years age group showed a growing trend in Brazil and all regions.

**Table 2:** Trend of fetal death numbers according to maternal age group (years). Brazil and regions. 1996–2015.

Regions	10–14		15–19		20–24		25–29		30–34		35–39		40–44		45–49		50–54		55–59		Age unknown	
	b	p	b	p	b	p	b	p	b	p	b	p	b	p	b	p	b	p	b	p	b	p
North	0.023	0.004^a^	0.009	0.159^b^	0.009	0.206^b^	0.017	0.042^a^	0.025	0.000^a^	0.024	0.000^a^	0.018	0.021^a^	0.001	0.904^b^	0.003	0.771^b^	0.001	0.753^b^	-0.019	0.000^c^
Northeast	0.019	0.006^a^	0.015	0.083^b^	0.014	0.140^b^	0.026	0.006^a^	0.032	0.001^a^	0.033	0.002^a^	0.025	0.017^a^	0.023	0.046^a^	0.011	0.070^b^	0.030	0.794^b^	-0.012	0.136^b^
Southeast	0.005	0.048^a^	-0.003	0.224^b^	-0.002	0.441^b^	0.002	0.394^b^	0.006	0.022^a^	0.006	0.079^b^	0.007	0.010^a^	0.002	0.695^b^	0.002	0.832^b^	0.008	0.798^b^	-0.027	0.001^c^
South	0.002	0.612^b^	-0.002	0.477^b^	0.001	0.710^b^	0.004	0.248^b^	0.008	0.027^a^	0.005	0.329^b^	0.005	0.366^b^	-0.001	0.841^b^	-0.004	0.612^b^	0.002	0.893^b^	-0.024	0.000^c^
Midwest	0.011	0.160^b^	0.009	0.113^b^	0.010	0.139^b^	0.018	0.015^a^	0.027	0.000^a^	0.030	0.000^a^	0.030	0.000^a^	0.033	0.010^a^	0.003	0.291^b^	0.005	0.938^b^	-0.020	0.000^c^
Brazil	0.012	0.001^a^	0.004	0.363^b^	0.005	0.361^b^	0.011	0.014^a^	0.015	0.000^a^	0.014	0.004^a^	0.013	0.016^a^	0.007	0.284^b^	0.018	0.312^b^	0.002	0.522^b^	-0.023	0.000^c^

a Increasing.

b Steady.

c Decreasing.

Mothers’ schooling in all regions was an important variable, with a reduction in the proportion of fetal deaths for mothers with up to seven years of schooling and an increase for mothers with more than eight years of schooling. The proportion of deaths for mothers whose level of education was unknown increased only in the Southeast region and in Brazil as a whole (Table 3).

**Table 3:** Percentage of fetal deaths according to maternal schooling. Brazil and regions, 1996–2015.

Regions	Schooling in 1996						Schooling in 2015					
	No	1–3 years	4–7 years	8–11 years	12 years or more	Unknown	No	1–3 years	4–7 years	8–11 years	12 years or more	Unknown
North	2.87	8.67	52.59	11.58	1.33	22.96	5.83	8.40	24.26	40.97	7.12	13.42
Northeast	14.85	16.61	34.93	10.21	2.17	21.23	4.10	10.00	26.11	37.06	7.19	15.54
Southeast	35.84	4.50	39.18	9.19	3.08	8.21	1.82	5.29	19.37	42.98	10.71	19.83
South	13.99	7.54	45.17	8.94	2.53	21.83	2.13	5.46	20.82	43.19	12.53	15.87
Midwest	15.20	5.74	46.73	8.65	3.31	20.37	3.39	4.56	21.40	41.17	13.72	15.76
Brazil	25.64	7.45	40.64	9.50	2.72	14.05	3.18	7.19	22.50	40.64	9.55	16.94

Table 4 summarizes data on the duration of gestation and types of delivery. Regarding the duration of gestation, fetal deaths occurred predominantly between the 28^th^ and 36^th^ weeks. There was an increasing trend of fetal deaths at this gestational age in Brazil and in all regions, except in the South (steady). There was a trend of decrease in fetal deaths at gestational age of 42 weeks or more in Brazil and in all regions. Vaginal delivery was the prevalent type of delivery in the country. Over the 19 years in Brazil, there was a steady trend for vaginal delivery (b = 0.006, p = 0.201) and an increasing trend (b = 0.014, p = 0.007) for cesarean sections.

**Table 4:** Trend of fetal death numbers according to duration of gestation and type of delivery. Brazil and regions, 1996–2015.

Variable			North		Northeast		Southeast		South		Midwest		Brazil	
			b	p	b	p	b	p	b	p	b	p	b	p
Duration of gestation														
	Less than 22 weeks		0.078	0.000^a^	0.086	0.000^a^	0.009	0.128^b^	0.054	0.000^a^	0.064	0.000^a^	0.031	0.000^a^
		22–27 weeks	0.068	0.015^a^	0.082	0.012^a^	0.007	0.265^b^	0.040	0.081^b^	0.065	0.012^a^	0.016	0.065^b^
		28–31 weeks	0.127	0.035^a^	0.151	0.031^a^	0.045	0.035^a^	0.067	0.124^b^	0.124	0.028^a^	0.060	0.030^a^
		32–36 weeks	0.064	0.034^a^	0.076	0.020^a^	0.070	0.044^a^	0.059	0.064^b^	0.070	0.034^a^	0.076	0.034^a^
		37–41 weeks	0.013	0.054^b^	0.022	0.019^a^	0.010	0.015^a^	-0.005	0.012^c^	0.012	0.003^a^	0.014	0.020^a^
		42 weeks or more	-0.044	0.000^c^	-0.039	0.000^c^	-0.072	0.000^c^	-0.080	0.000^c^	-0.063	0.000^c^	-0.054	0.000^c^
		Unknown	-0.012	0.437^b^	-0.021	0.090^b^	-0.002	0.046^c^	-0.022	0.033^c^	-0.036	0.043^c^	-0.026	0.083^b^
	Type of delivery													
		Vaginal	0.009	0.126^b^	0.016	0.061^b^	0.001	0.718^b^	-0.001	0.733^b^	0.010	0.072^b^	0.006	0.201^b^
		Ceasarean	0.024	0.003^a^	0.035	0.004^a^	0.005	0.048^a^	0.010	0.030^a^	0.021	0.002^a^	0.014	0.007^a^
		Unknown	-0.040	0.000^c^	-0.025	0.035^c^	-0.053	0.000^c^	-0.051	0.000^c^	-0.016	0.071^b^	-0.047	0.000^c^

a Increasing.

b Steady.

c Decreasing.

## DISCUSSION

The findings of this study indicate a steady trend in the country and in its regions, but there is still a long way to reach ideal levels, such as those of developed countries.

It is important to emphasize that the analysis of FMR in Brazil as a whole does not reflect the reality of each region, since the different social realities that directly impact on the numbers of deaths must be considered.

The Global Burden of Diseases^10^ (GBD) estimated age-specific and sex-specific mortality between 1970 and 2016 for 195 countries and territories, as well as the subnational level for five countries with a population greater than 200 million by 2016. The results of stillbirths per 1,000 live births showed the following estimates: 4.5 (4.3–4.6) in Central Europe, Eastern Europe and Central Asia; 6.3 (5.9–6.8) in Latin America and the Caribbean; 6.7 (6.4–7.0) in Oceania, East Asia and Southeast Asia; 10.4 (9.6–11.5) in North Africa and the Middle East; 17.4 (16.7–18.1) in South Asia; and, finally, 21.3 (20.2–22.6) in sub-Saharan Africa. From 2000 to 2016, the Brazilian fetal death rate was 5.3 (ranging from 4.9 to 5.8)^10^.

It is noteworthy that although the Brazilian FMR has shown a steady curve in this study, as the five regions of the country are analyzed by variables of interest (ICD, maternal age and education, duration of gestation and type of delivery), the findings deserve a deeper look and clarify the measures to be taken. The Figure shows very low fetal death rates from 1997 to 1999, which may be explained by the improvement in the information system.

Of the ICD-10 chapters, chapter XVII should be highlighted as it presented a growing curve in Brazil and in all regions. Chapter XVI has shown a growing trend only in the Northeast region. A systematic review of fetal deaths in Brazil^5^ mentions that assessing the underlying causes is frustrating, since the percentage of ill-defined causes is high and concordance is low. In addition, most of the studies raised in this review did not even specify whether the event occurred before or during delivery, which indicates limitations. Restricted intrauterine growth and maternal causes (hypertensive diseases, diabetes, syphilis, among others) were pointed out in the causal chain of fetal death. According to the list of preventable causes of death through SUS interventions^17^, avoidability is an indicator of effectiveness of health care and can direct managers to resources in improving prenatal and delivery care^5^.

Another important indicator of fetal mortality is maternal age. In the present study, we observed that women aged 25 to 44 years showed a growing curve in Brazil. Several studies^18^ ^-^ ^21^ corroborate this result; the older the mother, the higher the FMR. The North, Northeast and Midwest regions lead the growing trend of fetal mortality in the 25–44 age group, demonstrating that these regions need reinforcement in health care policies, such as improving the quality of prenatal, childbirth and puerperium care; control of sexually transmitted diseases and cervical and uterine cancer; conception and contraception care; improvement of obstetric care; family planning; attention to unsafe abortion and fight against domestic and sexual violence; and humanized care for pregnancy, childbirth and puerperium, as well as ensuring children the right to safe birth and healthy growth and development^11^ ^-^ ^14^.

The FMR is highly sensitive to check prenatal quality and intrapartum monitoring and care^22^. However, the available indicators capture only access to health services, not their effectiveness and quality of interventions. Empowering women has an important role in reducing fetal mortality rates, as they become capable of maximizing their health care, enabling family planning, prenatal care and quality intrapartum care^23^. One of the indicators that can contribute to this empowerment is women's mean years of schooling.

In this study, in relation to mothers’ schooling, fetal deaths in Brazil showed an increasing trend in two extremes: women with many years of schooling (eight or more) and consequently older (30 to 44 years) and adolescents (10 to 14 years of age), both outside the range of the reproductive peak. Risk pregnancies due to age stand out in these age groups. This may explain the finding of higher fetal mortality in women of higher educational level, a controversial result as compared to other studies^20^ ^,^ ^21^, in which low schooling was associated with fetal death.

There was a predominance of fetal death from the 28^th^ to the 36^th^ gestational week. This aspect is little explored in the national and international literature and was not a determinant of fetal death, probably due to difficulty in measuring the variable or absence in vital statistics.

Regarding type of delivery, although vaginal delivery prevails in the country, there is an increasing trend of cesarean sections in Brazil and regions for the period in question. An integrative review study on the practice of cesarean section in Brazil^24^ described the rates as abusive, alarming and worrying, forming a true epidemic, “a public health problem”. Cesarean birth compared to vaginal delivery is associated with an increase in maternal and neonatal morbidity and mortality in the short and long term^25^.

In Brazil, the determination of the FMR is a challenge that must be overcome in order to present methodological indicators comparable to those of countries with complete vital statistics. Despite the availability of the information system on deaths at the national level, several studies^26^ ^-^ ^28^ point to weaknesses in the quality of the information available for investigation of fetal deaths. One of the problems that still permeate the analyses of mortality in Brazil is the significant underreporting of deaths^5^.

Estimates, based on specific demographic procedures, can present methodological difficulties and inaccuracies inherent in the techniques used. Knowing the epidemiology of fetal death is extremely important for the promotion of actions geared to maternal and child health and also to support the adoption of preventive measures that allow a more effective confrontation of an avoidable problem^5^. Quality of information on fetal deaths and investments in research committees should be prioritized to reduce the FMR in Brazil. This investigation of fetal deaths in Brazil from 1996 to 2015 allows us to affirm that we need to pay more attention to the subject and to develop more valid epidemiological studies to increase its visibility.

Fetal mortality reflects the state of women's health, the quality and accessibility of primary health care provided to pregnant women, and the quality of intrapartum care^29^ ^,^ ^30^. It is considered a subject rarely studied in the Brazilian literature and statistics due to its low visibility, interest and negligence of the health services, which have not yet incorporated the analysis of its occurrence in work routines; specific investments for its reduction with public health policies and programs^3^ ^,^ ^8^ are also lacking.

In light of this information, we emphasize and reinforce the importance of high-quality prenatal and intrapartum care, continuing education of physicians and multiprofessional staff, and investments in fetal death investigation committees to reduce their rates in Brazil.

## Footnotes

a Chapter I: Certain infectious and parasitic diseases; II: Neoplasms; III: Diseases of the blood and blood-forming organs and certain disorders involving the immune mechanism; IV: Endocrine, nutritional and metabolic diseases; V: Mental and behavioral disorders; VI: Diseases of the nervous system; VII: Diseases of the eye and adnexa; VIII: Diseases of the ear and mastoid process; IX: Diseases of the circulatory system; X: Diseases of the respiratory system; XI: Diseases of the digestive system; XII: Diseases of the skin and subcutaneous tissue; XIII: Diseases of the musculoskeletal system and connective tissue; XIV: Diseases of the genitourinary system; XVI: Certain conditions originating in the perinatal period; XVII: Congenital malformations, deformations and chromosomal abnormalities; XVIII: Symptoms, signs and abnormal clinical and laboratory findings, not elsewhere classified; XIX: Injury, poisoning and certain other consequences of external causes; XX: External causes of morbidity and mortality; XXI: Contact with health services; XXII: Codes for special purposes

b Considering the underreporting of fetal deaths and the precariousness of available information on the duration of gestation, it is recommended to add both the numerator and the denominator the number of fetal deaths with unknown or incomplete gestational age. It is recommended to calculate this indicator only for the units of the Federation where the infant mortality rate is calculated by the direct method.
